# Educational interventions for general practitioners to identify and manage depression as a suicide risk factor in young people: a systematic review and meta-analysis protocol

**DOI:** 10.1186/2046-4053-3-145

**Published:** 2014-12-15

**Authors:** Lynda Tait, Maria Michail

**Affiliations:** School of Health Sciences, University of Nottingham, Nottingham, NG7 2HA UK; School of Health Sciences, Jubilee Campus, University of Nottingham, Nottingham, NG7 2TU UK

**Keywords:** Systematic review, Meta-analysis, Suicide, Prevention, Adolescents, Young adult, General practice

## Abstract

**Background:**

Suicide is a major public health problem and globally is the second leading cause of death in young adults. Globally, there are 164,000 suicides per year in young people under 25 years. Depression is a strong risk factor for suicide. Evidence shows that 45% of those completing suicide, including young adults, contact their general practitioner rather than a mental health professional in the month before their death. Further evidence indicates that risk factors or early warning signs of suicide in young people go undetected and untreated by general practitioners. Healthcare-based suicide prevention interventions targeted at general practitioners are designed to increase identification of at-risk young people. The rationale of this type of intervention is that early identification and improved clinical management of at-risk individuals will reduce morbidity and mortality. This systematic review will synthesise evidence on the effectiveness of education interventions for general practitioners in identifying and managing depression as a suicide risk factor in young people.

**Methods/design:**

We shall conduct a systematic review and meta-analysis following the Cochrane Handbook for Systematic Reviews of Interventions guidelines and conform to the reporting guidelines of the Preferred Reporting Items for Systematic Reviews and Meta-Analyses (PRISMA) statement recommendations. Electronic databases will be systematically searched for randomised controlled trials and quasi-experimental studies investigating the effectiveness of interventions for general practitioners in identifying and managing depression as a suicide risk factor in young people in comparison to any other intervention, no intervention, usual care or waiting list. Grey literature will be searched by screening trial registers. Only studies published in English will be included. No date restrictions will be applied. Two authors will independently screen titles and abstracts of potential studies. The primary outcome is identification and management of depression. Secondary outcomes are suicidal ideation, suicide attempts, deliberate self-harm, knowledge of suicide risk factors and suicide-related behaviours, attitudes towards suicide risk and suicide-related behaviours, confidence in dealing with suicide risk factors and suicide-related behaviour.

**Discussion:**

Our study will inform the development of future education interventions and provide feasibility and acceptability evidence, to help general practitioners identify and manage suicidal behaviour in young people.

**Systematic review registration:**

PROSPERO registration number: CRD42014009110.

**Electronic supplementary material:**

The online version of this article (doi:10.1186/2046-4053-3-145) contains supplementary material, which is available to authorized users.

## Background

Suicide is a major public health problem and globally is the second leading cause of death in young adults [1]. Suicide rates among males aged 15–24 years are estimated at 20 per 100,000 and for females 4.9 per 100,000 [2]. Globally, there are 164,000 suicides per year in young people under 25 years [3].

### Risk factors and early warning signs of suicide

Depression is consistently reported among those factors increasing the risk of death by suicide [4–7]. Substance abuse is also a significant risk factor, particularly among young people [8, 9]. Additional risk factors include a family history of suicide, being male, poor peer relationships, living apart from parents and traumatic events (e.g. sexual abuse) [4, 5, 10]. Parasuicide phenomena, including previous suicide attempts, deliberate self-harm, suicidal threats and thoughts, are positively associated with completed suicide [11, 12, 5].

### Suicide prevention programmes

Youth suicide prevention is a key public health target, and national strategies emphasise the importance of early identification, assessment and management of at-risk young people [1, 13]. Evidence-based interventions for suicide prevention fall into three categories [14]: 1) universal interventions target the entire population and aim to prevent suicide regardless of risk; 2) selective interventions target specific groups not showing signs of suicidal behaviour but who have risk factors that could increase future risk; and 3) indicated interventions target subgroups already showing suicidal behaviours such as deliberate self-harm and previous suicide attempts. Interventions within these three categories could take the form of school-based, family-focused and community-based suicide prevention programmes or healthcare prevention programmes that are aimed at general practitioners (GPs) and other primary care health workers [1, 15].

### Healthcare prevention programmes and the role of general practitioners

Considering that GPs are the primary point of contact for people with common mental health problems [16], they are key health professionals in a position to respond to and manage those who might be at risk of suicide. Evidence shows that 45% of those completing suicide, including young adults, contacted their GP rather than a mental health professional in the month before their death [17]. There is further evidence in the literature showing that risk factors or early warning signs of suicide in young people go undetected and untreated by GPs [18–20]. In a study examining the availability of services in primary care for the management of suicidal patients [21], GPs reported concerns about the mental health training they receive, specifically with regard to the prevention of self-harm and suicide. Providing GPs with adequate training and education could lead to increased identification of those at risk, which has the potential to be cost effective if it leads to adequate subsequent treatment and improves the mental health and well-being of young people. Indeed, gatekeeper training, including primary care health providers, has been highlighted as an important suicide prevention strategy [1, 15, 22]. Interventions focusing on awareness raising and education about suicide and risk factors (including depression) and also interventions focusing on training GPs on screening for at-risk individuals and clinical management are considered promising strategies [23].

These findings suggest the importance of training GPs to improve early identification and management of young people at risk of suicide. Previous studies have shown that multifaceted education interventions in primary care are likely to produce positive results [22, 24, 25].

### Healthcare-based suicide prevention interventions

Relevant preventive interventions for individual suicide risk factors (selective and indicated interventions) include the assessment and management of suicide behaviours and mental and substance use disorders [1]. Education interventions targeting healthcare professionals are designed to improve clinical skills needed to assess and manage suicide risk factors, including the effective treatment of mental health disorders such as depression. [1, 15, 26].

The logic underpinning education interventions focuses on training health professionals to improve the clinical skills required to identify and manage individuals at risk of suicide in order to reduce suicide risk factors. There is some evidence that training healthcare professionals may improve identification rates of patients at risk of suicide [5], and a systematic review concluded that physician education on depression recognition and treatment reduced suicide rates [23].

Two specific international training programmes include the Question, Persuade and Refer (QPR), and the Skills Training on Risk Management (STORM). The QPR [27] intervention is presented in an e-learning format and delivered in eight modules designed to teach recognition of someone at risk of suicide, how to ask direct questions about suicidal thoughts and plans and how to make timely referrals. The content of the STORM [28] training consists of the assessment of suicide risk, mental state and psychosocial problems, the clinical management of suicide risk and emotional crises and the prevention of further crises. Training, conducted over 6 h, includes written handouts, oral and video presentations, role play and discussion.

### The importance of this systematic review

Primary care presents one of the most appropriate pathways for the identification of, and early intervention for, suicide risk. This is because GPs are the first point of contact for people in distress [16], and are gatekeepers to specialist treatment services. This systematic review will retrieve and synthesise evidence on the effectiveness of education interventions for GPs in the identification and management of suicidal behaviour in young people. It will also provide evidence on the feasibility and acceptability of these interventions and delineate, if possible, which processes or mechanisms of the interventions are most successful in identifying and managing suicidal behaviour in young people. In doing so, this systematic review will provide recommendations for suicide prevention in primary care and will inform the development of future interventions. Available systematic reviews that focus on or include the role of primary care in youth suicide prevention are either outdated and have not included a meta-analysis [19, 20, 5, 23] or simply provide a scoping review of the literature [18].

### Aim

The aim of this systematic review and meta-analysis is to determine the effectiveness of education interventions for GPs in identifying and managing suicide risk in young people aged 14–25 years.

### Objectives

The objectives are:

1. To synthesise the evidence of the effectiveness of interventions for GPs in identifying and managing depression as a suicide risk factor in young people aged 14–25 years.
2. To estimate the aggregate effectiveness of interventions for GPs in identifying and managing depression as a suicide risk factor in young people aged 14–25 years.

## Methods/design

### Study design

We shall conduct a systematic review and meta-analysis following the Cochrane Handbook for Systematic Reviews of Interventions guidelines [29] and conform to the reporting guidelines of the Preferred Reporting Items for Systematic Reviews and Meta-Analyses (PRISMA) statement recommendations [30].

### Search strategy

The literature search strategy is included in Additional file 1. The following search limits will be set: 1) study design: randomised controlled trials (RCTs) and quasi-experimental studies (controlled before-after studies, i.e. pre- and post-test designs), 2) limited to English language. No date restrictions will be applied. The following bibliographic databases will be searched:

- ● Cochrane Central Register of Controlled Trials
- ● CINAHL (Cumulative Index to Nursing and Allied Health Literature)
- ● Embase
- ● MEDLINE
- ● PsycINFO
- ● SCI (Science Citation Index)

### Grey literature

- ● Clinical Trials: http://clinicaltrials.gov
- ● ISRCTN Register

### Search terms

We shall use Medical Subject Headings (MeSH) and free-text word terms, as appropriate to the databases. To optimise our search strategies to identify RCTs, we shall use the clinical search filters for electronic databases, which are recommended by York University [31] and SIGN [32]. Search fields are: OVID all fields; SCI topic; CINAHL text and Cochrane title, abstract and keywords. The electronic search strategy terms (where necessary using wildcards) are:

MeSH terms included:

- General Practice, Primary Care, Family practice Primary Health Care;
- Intervention, Education, Training, Screening;
- Suicide, Suicide attempted, Suicidal ideation, Depression, Deliberate self-harm;
- Child, adolescent;
- Treatment efficacy, Treatment effectiveness.
- Gatekeeper training, suicidal thinking, suicide risk will be used as free-text word terms as they are not included within MeSH exploded terms.
- Randomized Controlled Trials, Clinical Trial, Random Allocation, Randomly Allocated, Double-Blind Method, Single-Blind Method, Randomized Controlled Trial, Multicentre Study.
- Search strategies will be pilot tested. We shall use EndNote (Thomson Reuters) to record titles and abstracts for retrieval and inclusion/exclusion decisions.

### Selection criteria

#### Types of participants

We will include studies of participants who are general practitioners and young people aged 14–25 years.

#### Types of trials

RCTs and quasi-experimental study designs (controlled before-after studies, i.e. pre- and post-test designs) will be included in the review. Control groups will be contemporaneous, with two time points. Non-RCTs are included because there was a limited number of RCTs found in our scoping searches. Systematic reviews, dissertations/theses or studies describing an intervention but not providing any evaluation will be excluded.

#### Types of intervention

The review will include selective interventions (targeting subgroups that are not showing signs of suicidal behaviour but that are displaying risk factors that could place them at greater risk in the future) and indicated interventions (targeting subgroups that are already displaying suicidal behaviours such as deliberate self-harm). These could include primary care/gatekeeper training programmes, educational interventions/training, identification and screening programmes.

#### Control condition

Control conditions will include any other intervention or no intervention/usual care or waiting list.

### Types of outcomes

#### Primary outcome

Eligible studies will have as primary outcome rates of identification and management of depressive disorders or depressive symptoms. There are substantial difficulties in demonstrating a direct link between a reduction in suicide rates and the effectiveness of suicide interventions, such as inaccuracy and the unreliability of suicide reports, the large number of potential confounding factors associated with suicide, that completed suicide is a rare event, and education interventions of interest will be targeted at health professionals and not at at-risk individuals. Therefore, eligible studies will have as primary outcome rates of identification and management of depressive disorders or depressive symptoms.

The aim of this review is to include studies that report on intermediate data that include depressive disorders or depressive symptoms.

Depression and depressive symptoms—included studies must have assessed depression or depressive symptoms using any validated self-reported or interviewer-administered scales (for example, the use of the Beck Depression Inventory for depression) [33]. We shall also include studies relevant to standard diagnostic criteria for the assessment of depression based on the International Classification of Diseases (ICD-10) definition of depression [34] and the DSM-IV definition [35].

#### Secondary outcomes

Secondary outcomes will include:

- suicidal ideation, referring to thoughts about or an unusual preoccupation with death, as assessed using self-reported or interviewer-administered scales (e.g. Suicidal Intent Scale [36]; The Modified Scale for Suicidal Ideation [37])
- suicide attempts, where there is clear intention to die, as assessed by using any self-reported or interviewer-administered scales (e.g. Suicidal Behaviours Questionnaire [38])
- deliberate self-harm, referring to self-destructive behaviours (e.g. cutting, poisoning), which reflect intentional, immediate and direct bodily damage where death is not necessarily the intended outcome [39–41], assessed using any self-reported or interviewer-administered scales (e.g. Deliberate Self-harm Inventory [42]; Inventory of Statements about Self-injury [43])
- GP knowledge of suicide risk factors and warning signs, as assessed using self-reported or interviewer-administered scales (e.g. Recognizing Suicide Lethality Scale [44])
- GP attitudes towards suicide risk and suicide-related behaviours, as assessed by self-reported or interviewer-administered scales such as the Attitudes towards Suicide Scale [45]
- confidence in dealing with suicide risk factors, and suicide-related behaviour, as assessed by self-reported confidence ratings [46].

### Selection procedure

Two researchers (LT and MM) will independently screen the title and abstract of retrieved references for inclusion. The full text of all potential eligible studies will be obtained by MM. The next step will involve two researchers (LT and MM) independently assessing obtained references against the eligibility criteria for inclusion. We shall pilot test the procedure on a small number of studies. Results will be compared and any disagreements will be resolved by discussion and consensus.

### Managing references

Bibliographic software (EndNote) (Thomson Reuters) will be used to manage retrieved references. MM will be responsible for identifying and removing duplicates, ordering and recording the receipt of any inter-library loans and obtaining the full-text papers. MM will be the sole team member responsible for adding or amending library records in EndNote.

### Data extraction, procedures and data management

The Effective Practice and Organisation of Care Review Group (EPOC) data abstraction form [47] in combination with the EPOC data collection checklist [48] will be used to extract data from relevant studies. Two reviewers (LT and MM) will work independently to extract data. Disagreements between the two reviewers will be resolved by discussion and consensus. Data extraction will include study setting, study population and participant demographics and baseline characteristics, details of the intervention and control conditions; study methodology, recruitment and study completion rates, outcomes and times of measurement; indicators of acceptability to users, suggested mechanisms of intervention action, information for assessment of the risk of bias and variables related to study quality.

### Dealing with missing data

We shall contact the original study authors to request missing data. Information on missing data and dropouts will be assessed and reported for each study. We shall calculate or estimate data that is missing from other statistics available. For example, in the case of missing standard deviations we shall use confidence intervals, standard errors, *F* values, *t* values and *P* values to calculate the missing standard deviation values, following rules described in the Cochrane Handbook for Systematic Reviews of Interventions (Chapters 7.7.3 and 16.1.3) [29]. If we cannot apply the formulae described in the Cochrane Handbook, missing values will be imputed according to a validated method and after consulting with a statistician.

We shall conduct a sensitivity analysis to examine the effects of any imputed missing values and how sensitive results are to changes in the assumptions we have made. The possible effects of missing data on the review findings will be addressed in our discussion section.

### Quality assessment

We shall assess the quality of studies and assessment of bias using the Cochrane’s Collaboration tool for assessing risk of bias [49]. Using the tool, we shall rank potential sources of bias for RCTs and non-RCTs into low, unclear or high risk of bias for each main outcome (across domains) within and across studies. Two researchers (LT and MM) will independently rate the risk of bias of each study. Discrepancies will be resolved by discussion and consensus.

### Data analysis

#### Measures of treatment effects

We shall summarise and describe study characteristics of the population, interventions and outcomes, using descriptive statistics. Effect sizes expressed as weighted mean differences or standardised mean differences (where studies have assessed the same outcome but have used different measures, for example, different scales to measure depression), Hedges *g*, and weighting studies using inverse of variance and their 95% confidence intervals (CI), will be calculated for continuous outcomes. For dichotomous data, risk ratios (RR) with 95% CI will be calculated. We shall use the Mantel-Haenszel method to combine studies, where appropriate. We shall report outcomes using 95% CI with random-effects models.

### Assessment of reporting biases

Funnel plots will be drawn to investigate the relationship between study power and effect size. Possible reasons for any asymmetry will be discussed.

### Data synthesis and assessment of heterogeneity

Evidence from RCTs and non-RCTs will not be combined but will be presented separately. A meta-analysis of RCTs will be conducted using RevMan. For the meta-analyses, we shall pool data using a random-effects model to allow for differences between studies, although there is no statistically significant heterogeneity. Effect estimates will be weighted by the inverse of their variance, giving greater weight to larger trials. For non-RCTs, we shall carefully consider the following before deciding to conduct a meta-analysis: a) weakness in the study design, b) execution of study following a risk of bias assessment, c) selection bias and confounding, and d) potential reporting biases. If a meta-analysis is deemed inappropriate, we shall provide a narrative synthesis following guidance [50].

We shall visually inspect graphs to examine possible statistical heterogeneity between studies. Statistical tests of heterogeneity (chi-square *P* value and *I*-square statistics) will also be carried out. If we find substantial levels of heterogeneity, reasons will be explored using sub-group analyses.

We shall carry out sub-group analyses to identify whether there are differences in rates of detection of suicide risk between educational programmes only versus educational programmes that incorporate screening training versus screening only training for GPs.

Sensitivity analysis will be performed to assess how robust the synthesis is to the addition or removal of the lowest quality studies (i.e. for RCT studies where allocation concealment has not been undertaken or not clearly stated as undertaken, and studies with more than a 50% drop-out rate; for non-RCT studies where there is incomplete outcome data, selective reporting or bias related to specific study design).

We shall use the Grading of Recommendations Assessment and Development and Evaluation (GRADE) system 11 [51] to assess confidence in the quality of evidence of individual outcomes and strength of recommendations.

## Discussion

Youth suicide prevention is a key public health target, with national strategies highlighting the importance of early identification, assessment and management of at-risk young people. Healthcare prevention programmes encourage the involvement of GPs, but caring for young people at risk of suicide requires specialist knowledge and skills that GPs are reportedly concerned about. Suicide prevention education for GPs may therefore be an important step in improving suicide prevention in primary care. Available systematic reviews that focus on, or include, the role of primary care in youth suicide prevention are either outdated and have not included a meta-analysis or simply provide a scoping review of the literature.

This systematic review synthesising evidence on the effectiveness of education interventions for GPs in the identification and management of suicidal behaviour in young people will provide an important contribution to the literature on suicide prevention in primary care. It will also provide evidence on the feasibility and acceptability of those interventions and delineate, if possible, which processes or mechanisms of the interventions are most successful in identifying and managing suicidal behaviour in young people. This systematic review will therefore provide recommendations for suicide prevention in primary care and inform the development of future education interventions.

### Defining terms

‘Young people’ and ‘youth’

Refer to the age group in our study (14–25 years), which includes ‘adolescents’ (aged 14–19 years).

## Electronic supplementary material

Additional file 1: **Literature search strategy.** The file provides a description of the literature search strategy. (DOCX 27 KB)

## Abbreviations

*GPs*: general practitioners
*PRISMA*: Preferred Reporting Items for Systematic Reviews and Meta-Analyses
*RCTs*: Randomised controlled trials
*MeSH*: Medical Subject Headings
*EPOC*: Effective Practice and Organization of Care
*RR*: risk ratio
*CI*: confidence interval
*OR*: odd ratios.
