# Supplementary material for: Educational interventions for general practitioners to identify and manage depression as a suicide risk factor in young people: a systematic review and meta-analysis protocol
Source: Syst Rev. 2014 Dec 15;3:145. doi: 10.1186/2046-4053-3-145 (PMC4276044; doi:10.1186/2046-4053-3-145)
Supplement: Supplementary file 1 — Additional file 1: Literature search strategy. The file provides a description of the literature search strategy. (DOCX 27 KB) [file 13643_2014_312_MOESM1_ESM.docx]

**Additional file 1: Literature search strategy (highly sensitive search strategy)**

| Search terms (AND, OR, NOT) and truncation (wildcard characters like *) | 1. exp Family Practice/or exp General Practice/ 2. exp Primary Health Care/ 3. (general practice* or primary care or family practice*).af. 4. exp “Early Intervention (Education)”/or exp Intervention Studies/or exp Early Medical Intervention/ 5. exp Education/ 6. (intervention* or education* or “gatekeeper train*” or screen* or identif*).af. 7. exp Suicide/or exp Suicide, Attempted/ or exp Suicide, Assisted/ 8. exp Depression/ 9. (suicide* or “suicide* attempt” or “suicide* ideation” or “suicide* thinking” or “suicide* risk” or depress* or “deliberate self harm”).af. 10. exp Adolescent/ 11. exp Young Adult/ 12. (youth or “young adult” or “young people” or teenager* or teens or adolescen*).af. 13. 1 or 2 or 3 14. 4 or 5 or 6 15. 7 or 8 or 9 16. 10 or 11 or 12 17. 13 and 14 18. 15 and 17 19. 16 and 18 20. exp Randomized Controlled Trials as Topic. 21. exp Clinical Trial/ 22. exp Random Allocation/ 23. exp Double-Blind Method/ 24. exp Single-Blind Method/ 25. exp Randomized Controlled Trial/ 26. (clinical trial, phase i or clinical trial, phage ii or clinical trial, phase iii or clinical trial, phase iv).pt. 27. (randomized controlled trial or multicentre study or clinical trial).pt. 28. exp clinical trials as Topic 29. 20 or 21 or 22 or 23 or 24 or 25 or 26 or 27 or 28 30. ((clinical adj trial*) or placebo* or randomly allocated or (allocated adj2 random*)).tw. 31. (singl* or doubl* or treb* or tripl*) adj (blind$3 or mask*)).tw. 32. exp Placebos/ 33. 30 or 31 or 32 34. 29 or 33 35. 19 and 34 36. (efficacy* or effect*).mp. [mp=title, abstract, original title, name of substance word, subject heading word, keyword heading word, protocol supplementary concept word, rare disease supplementary concept word, unique identifier] 37. 35 and 36 38. Limit 37 to English language 39. Case-report.tw. or letter/or historical-article/ 40. 38 not 39 |
| --- | --- |
| Databases searched | *All relevant databases for the topic area such as: CINAHL, Medline, Proquest Central*  Cochrane Central Register of Controlled Trials  CINAHL (Cumulative Index to Nursing and Allied Health Literature)  EMBASE  MEDLINE  PsychINFO  SCI (Science Citation Index)  **Grey Literature**  Clinical Trials: [clinicaltrials.gov](http://clinicaltrials.gov)  ISRCTN Register |
| Part of journals searched | *Did you use: keywords in abstract and title, subject headings and so on?*  Subject headings (MeSH) and free-text word terms will be used to screen abstracts and titles |
| Years of search | *This depends on the amount of ongoing research published in journals. E.g. 2001-2011*  Date restrictions will not be applied |
| Language | *E.g. English*  English language only |
| Types of studies to be included | *E.g. qualitative studies*  Randomised controlled trials  Quasi-experimental studies (controlled before and after, i.e. pre- and post-test design). |
| Inclusion criteria (why did you include it?) | *Type of participants*   1. General Practitioners 2. Young people aged >14yrs   *Type of interventions*  Selective interventions (targeting subgroups that are not showing signs of suicidal behaviour but that are displaying risk factors that could place them at greater risk in the future) and indicated interventions (targeting subgroups that are already displaying suicidal behaviours such as deliberate self-harm). These could include primary care/gatekeeper training programs, educational interventions/training, identification and screening programs.  *Type of outcomes*  Primary Outcome: Depression and depressive symptoms.  Secondary Outcomes: Suicidal ideation; suicide attempts; deliberate self-harm; GP knowledge of suicide risk factors, and warning signs; GP attitudes towards suicide risk and suicide-related behaviours; attitudes towards suicide risk and suicide related behaviours; confidence in dealing with suicide risk factors and suicide-related behaviour*.* |
| Exclusion criteria (why did you rule it out? | Systematic reviews, dissertations/theses or studies describing an intervention but not providing any evaluation will be excluded |
